# Supplementary material for: Comprehensive Profiling of Alternative Splicing and Alternative Polyadenylation during Fruit Ripening in Watermelon (Citrullus lanatus)
Source: Int J Mol Sci. 2023 Oct 18;24(20):15333. doi: 10.3390/ijms242015333 (PMC10607834; doi:10.3390/ijms242015333)
Supplement: Supplementary file 1 [file ijms-24-15333-s001.zip › Supplementary Information.pdf]

Supplementary data

Yu *et al*., Comprehensive profiling of alternative splicing and alternative polyadenylation during fruit ripening in watermelon (*Citrullus lanatus*)

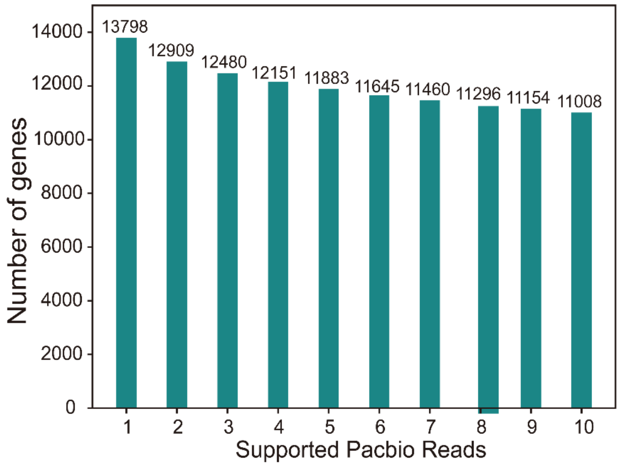

Supplementary Figure S1. Number of genes with supported PacBio reads.

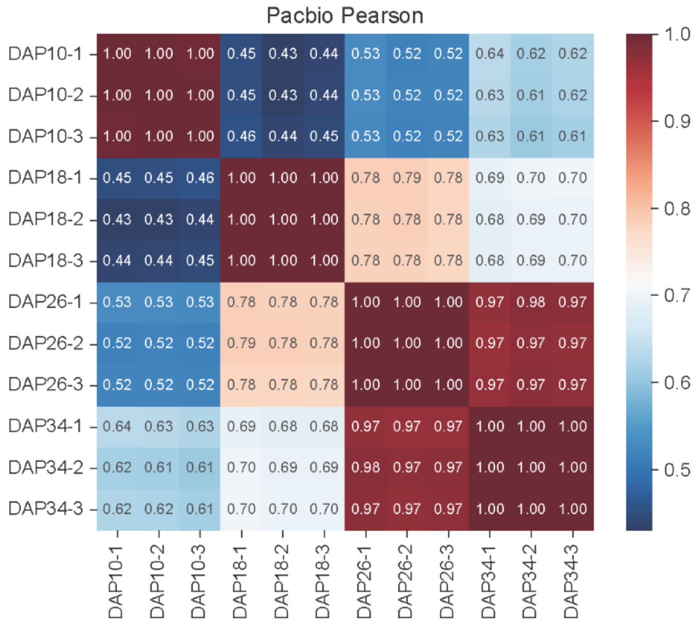

Supplementary Figure S2. Heatmaps of Pearson correlation values of 12 samples.

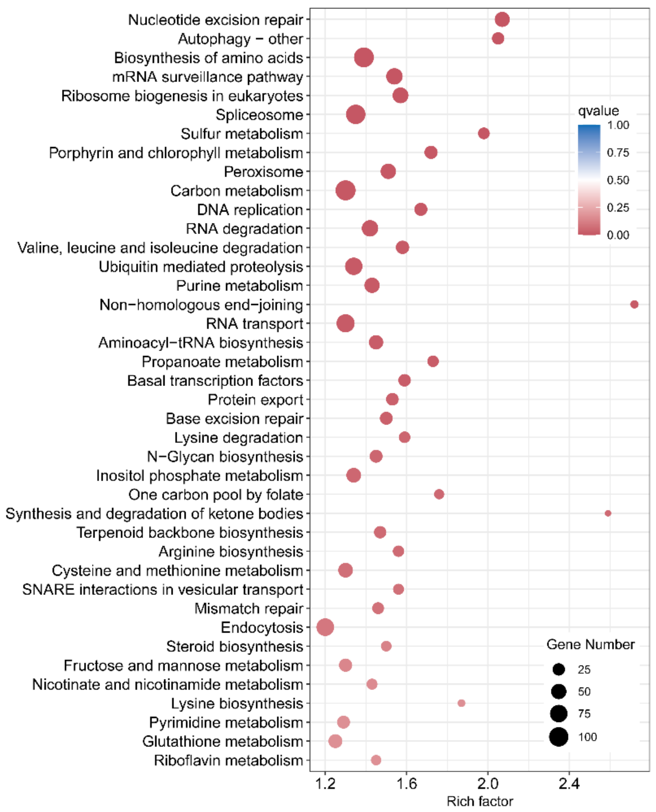

Supplementary Figure S3. KEGG analysis of genes undergoing AS.

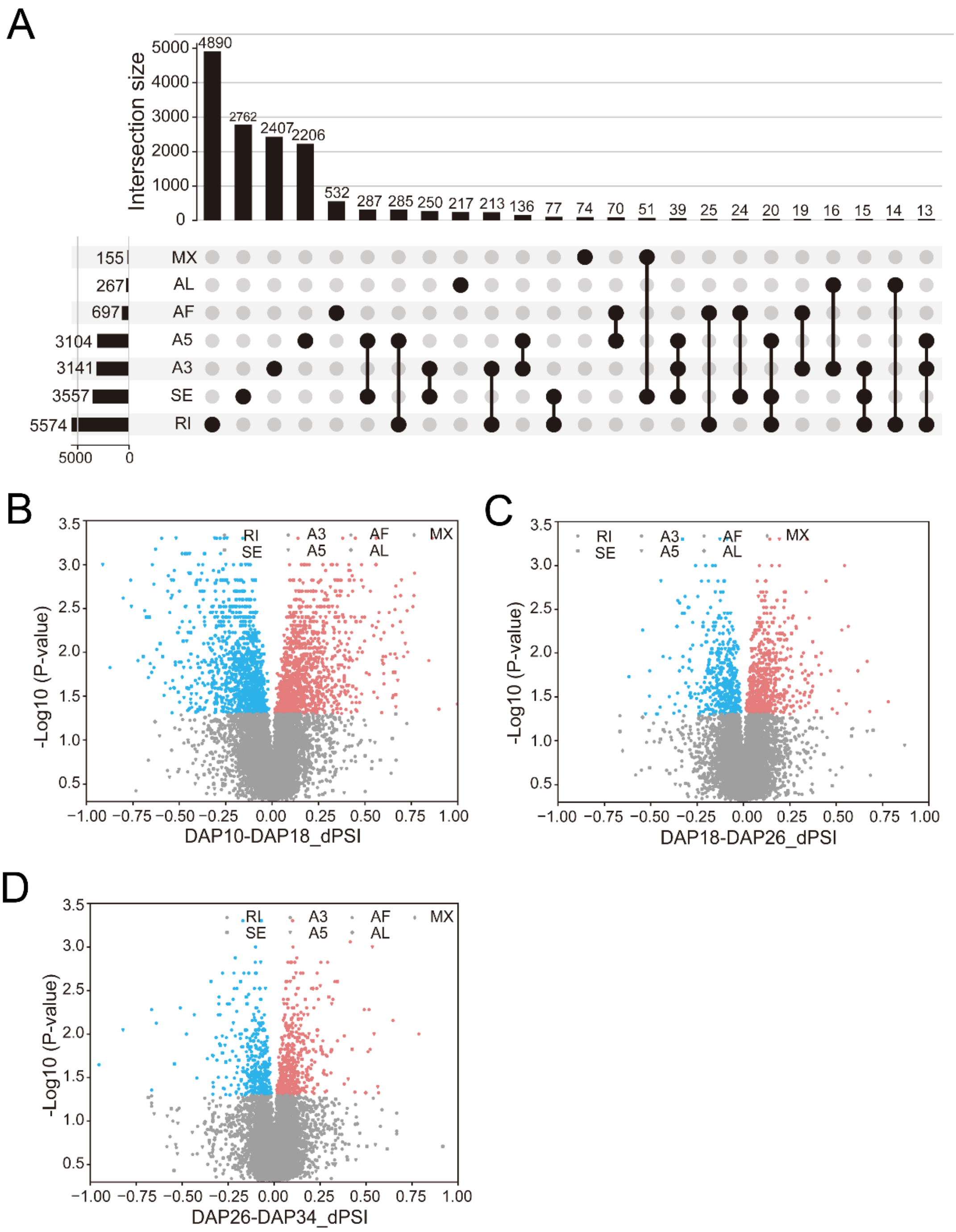

Supplementary Figure S4. Statistics of AS events and quantification of AS in watermelon fruit. (A): Statistics of the 7 types of AS events. (B)-(D): Quantification of differential alternative splicing in DAP 18 vs DAP 10 (B), DAP 26 vs DAP 18 (C) and DAP 34 vs DAP 26 (D).

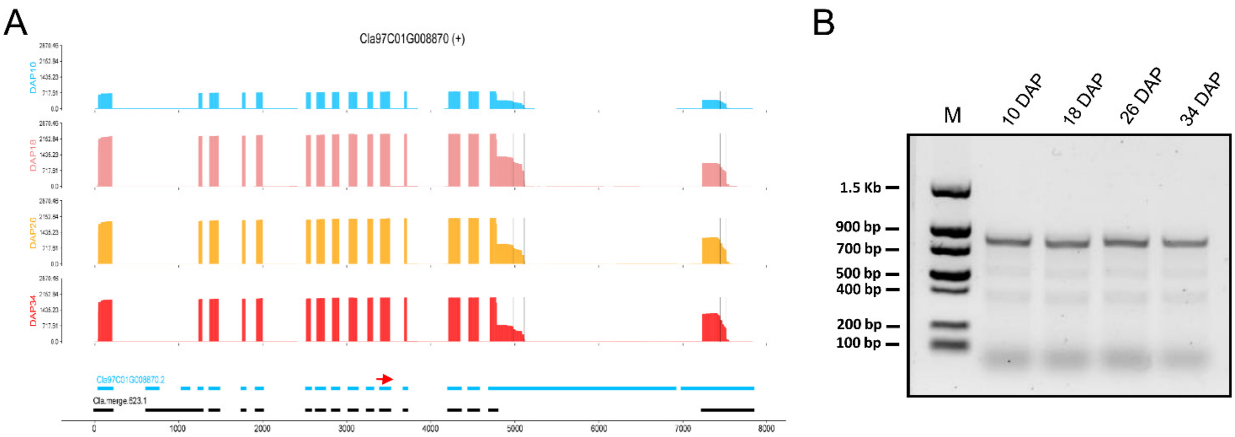

Supplementary Figure S5. Validation of APA. (A): Wiggle plot showed that Cla97C01G008870 (ICDH) displaed differential alternative polyadenylation sites. The red arrow represents the position of forward primer. (B): Validation of alternative polyadenylation by 3' rapid amplification of cDNA ends (3'-RACE).

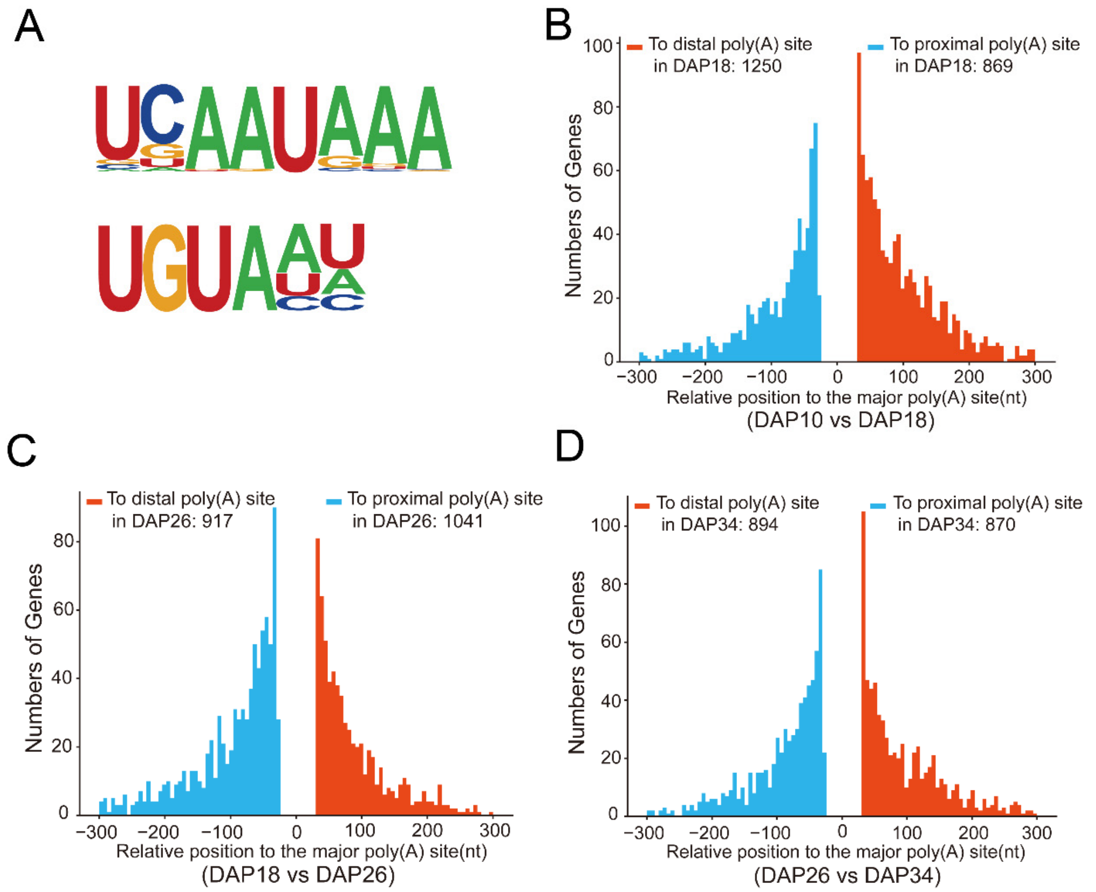

Supplementary Figure S6. Identification of motifs around polyadenylation sites and quantification of differential alternative polyadenlation. (A): Two motifs (AAUAAA, UGUA) are found near the polyadenylation site. (B)-(D): Quantification of differential alternative polyadenlation in three groups (DAP 18 vs DAP 10, DAP 26vs DAP 18 and DAP 34 vs DAP 26).

Supplementary Table S1. Primers used for validation of AS and APA

**Validation of AS (Cla97C02G037750)**<br>forward primer:5'- AACGAGCTGTTGCCGCTAAATGATGAA-3'<br>reverse primer:5'- GTTTATGATCCAGTCTCCAGCCATGAACA-3'
**Validation of APA (Cla97C01G008870)**<br>forward primer:5'-ATATTCCAAGAAGTCTATGAATCCCA-3'<br>reverse primer:5'-TACCGTCGTTCCACTAGTGATTT-3'

Supplementary Data S1. Identification of AS events in watermelon

Supplementary Data S2. KEGG analysis of genes undergoing AS

Supplementary Data S3. AS quantification in three groups

Supplementary Data S4. Global APA sites in watermelon

Supplementary Data S5. KEGG analysis of genes undergoing APA

Supplementary Data S6. APA quantification in three groups

Supplementary Data S7. Total high-confident lncRNAs in watermelon fruit.

Supplementary Data S8. LncRNAs quantification in three groups
